# Early MRI Predictors of Relapse in Primary Central Nervous System Lymphoma Treated with MATRix Immunochemotherapy

**DOI:** 10.3390/jpm13071182

**Published:** 2023-07-24

**Authors:** Isabel Cornell, Ayisha Al Busaidi, Stephen Wastling, Mustafa Anjari, Kate Cwynarski, Christopher P. Fox, Nicolas Martinez-Calle, Edward Poynton, John Maynard, Steffi C. Thust

**Affiliations:** 1 UCL Institute of Neurology, Department of Brain Rehabilitation and Repair, Queen Square, London WC1N 3BG, UKmustafa.anjari@nhs.net (M.A.);; 2 Lysholm Department of Neuroradiology, National Hospital for Neurology and Neurosurgery, University College London Hospitals NHS Foundation Trust, London WC1N 3BG, UK; 3 Neuroradiology Department, Kings College Hospital NHS Foundation Trust, London SE5 9RS, UK; 4 Radiology Department, Royal Free London NHS Foundation Trust, London NW3 2QG, UK; 5 Haematology Department, University College London Hospitals NHS Foundation Trust, London NW1 2BU, UK; 6 School of Medicine, University of Nottingham, Nottingham NG7 2UH, UK; christopher.fox@nottingham.ac.uk (C.P.F.);; 7 Precision Imaging Beacon, School of Medicine, University of Nottingham, Nottingham NG7 2UH, UK; 8 Neuroradiology Department, Nottingham University Hospitals NHS Trust, Nottingham NG7 2UH, UK

**Keywords:** CNS lymphoma, MATRix, response imaging

## Abstract

Primary Central Nervous System Lymphoma (PCNSL) is a highly malignant brain tumour. We investigated dynamic changes in tumour volume and apparent diffusion coefficient (ADC) measurements for predicting outcome following treatment with MATRix chemotherapy in PCNSL. Patients treated with MATRix (*n* = 38) underwent T1 contrast-enhanced (T1CE) and diffusion-weighted imaging (DWI) before treatment, after two cycles and after four cycles of chemotherapy. Response was assessed using the International PCNSL Collaborative Group (IPCG) imaging criteria. ADC histogram parameters and T1CE tumour volumes were compared among response groups, using one-way ANOVA testing. Logistic regression was performed to examine those imaging parameters predictive of response. Response after two cycles of chemotherapy differed from response after four cycles; of the six patients with progressive disease (PD) after four cycles of treatment, two (33%) had demonstrated a partial response (PR) or complete response (CR) after two cycles. ADC_mean_ at baseline, T1CE at baseline and T1CE percentage volume change differed between response groups (0.005 < *p* < 0.038) and were predictive of MATRix treatment response (area under the curve: 0.672–0.854). Baseline ADC and T1CE metrics are potential biomarkers for risk stratification of PCNSL patients early during remission induction therapy with MATRix. Standard interim response assessment (after two cycles) according to IPCG imaging criteria does not reliably predict early disease progression in the context of a conventional treatment approach.

## 1. Introduction

Primary Central Nervous System Lymphoma (PCNSL) is a highly malignant brain tumour originating from lymphocytes, typically in the form of diffuse large B-cell lymphoma (DLBCL). The incidence of PCNSL is approximately 0.5/100,000 persons/year with a predilection for older individuals. The rising incidence of PCNSL over the last decades [1,2,3,4] and its responsiveness to chemotherapy have led to a growing focus on its management. Until recent years, PCNSL was often an incurable disease with long-term survival rates of approximately 20–30% [5]. Based on results of international randomised trials such as research conducted by the International Extranodal Lymphoma Study Group (IELSG), therapeutic strategies have evolved with a dramatic improvement in PCNSL outcomes. Since 2016, newly diagnosed PCNSL patients ≤70 years old with sufficient fitness are offered remission induction treatment consisting of four cycles of MATRix chemotherapy (methotrexate, cytarabine, thiotepa and rituximab) followed by High-Dose Therapy and Autologous Stem Cell Transplantation (HDT-ASCT), as established by IELSG32 [6]. The MATRix regimen is a highly effective treatment for PCNSL but carries a substantial risk of treatment-related neurotoxicity. Treatment-related mortality lies in range of 4–7% [7]. These complications preclude some patients from proceeding to HDT-ASCT consolidation, which likely represents an essential milestone towards survival. In addition, there are few long-term data on the side effects of MATRix; however, its components (specifically methotrexate) are known to be neurotoxic such that treatment-related neurocognitive deficits may have important and possibly underestimated long-term clinical impact. For patients with relapsed or refractory PCNSL, survival expectations are very limited with a median OS of 3.5 months [8]. It would be highly advantageous to confidently identify patients at high risk of treatment failure, earlier in the treatment pathway, towards risk-adapted treatment strategies.

Presently, the standard for imaging-based response assessment in PCNSL is based on recommendations by the International Primary CNS Lymphoma Collaborative Group (IPCG) [1] (Table 4). The standardised guidelines divide PCNSL patients receiving MATRix chemotherapy into four groups according to response, i.e., complete response (CR), unconfirmed complete response (CRu), partial response (PR) and progressive disease (PD), and are summarized in Table 1.

The radiological component of the IPCG categorical response criteria remains confined to a semi-qualitative (percentage estimate) lesion assessment based on gadolinium enhancement, despite increased clinical availability of quantitative imaging methods [4]. It has recently been suggested that IPCG-derived response has limited interobserver agreement and may not accurately predict long-term survival [9]. This is partially due to lack of adoption of standardized MRI acquisition parameters and sequences in PCNSL assessment, which has led to the IPCG suggesting ”ideal” and ”minimum” recommended PCNSL imaging protocols for both 3 T and 1.5 T MRI systems [10]. There are currently no established physiological imaging biomarkers to inform treatment approaches in PCNSL. In recent years, focus has shifted towards the possible diagnostic and prognostic role of quantitative imaging analysis in PCNSL [5], with the hope of finding correlates that reflect treatment efficacy and would be suitable for clinical translation [11]. As a first step, rather than the more clinically widespread use of two-dimensional sum-product measurements of tumour burden, contrast-enhancing lesion volumetry could provide a more accurate and objective parameter for response assessment [10].

Mean Apparent Diffusion Coefficient (ADC) measurements could be a rapid and practicable approach to improving imaging response assessment of PCNSL, which is widely known to display low diffusion (for example, [12]). ADC values are calculated from diffusion-weighted imaging (DWI) and represent a measure of the diffusion of water molecules in a given time period as a method for assessing tissue/tumoral microstructure. Typical PCNSL is characterised by a higher cellular density compared to many other CNS malignancies which corresponds to a lower ADC [5,13]. The use of ADC parameters has shown to be valuable for a number of applications, including PCNSL from glioblastoma distinction [14,15] and glioma molecular subgroup predictions. ADC measurement in PCNSL could represent a further step towards a more accurate, quantifiable image assessment.

The purpose of this research study was to test the hypothesis that baseline and early-in-therapy T1 contrast-enhanced (T1CE) lesion volume measurements and diffusion-weighted imaging (ADC values) cumulative parameters may allow prediction of response to MATRix chemotherapy in PCNSL.

## 2. Materials and Methods

### 2.1. Patients

**Figure 1:**
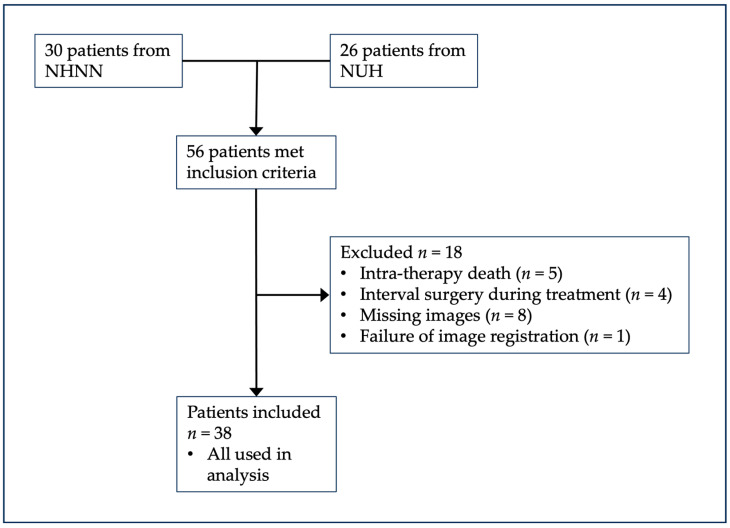
Patient inclusion and exclusion criteria.

Consecutive patients (*n* = 56) who received MATRix chemotherapy at two different treatment centres, the National Hospital of Neurology and Neurosurgery, University College London Hospitals NHS Foundation Trust (NHNN, *n* = 30), and Nottingham University Hospital (NUH, *n* = 26), were eligible for the analysis. Inclusion criteria comprised the following: age over 18 years, histological confirmation of primary DLBCL of the CNS as defined by the World Health Organisation Classification of CNS Tumours [16,17], treatment with MATRix chemotherapy, no evidence of systemic Non-Hodgkin Lymphoma, serology-negative for human immunodeficiency virus and available contrast-enhanced and diffusion-weighted imaging (DWI and ADC) at baseline, after 2 cycles of treatment and within one month of completion of MATRix treatment. Ethics review board approval (Health Research Authority, United Kingdom, IRAS 218180) was obtained with informed consent waived for this retrospective imaging data study. Exclusion criteria were intra-therapy death, leading to non-completion of treatment and, therefore, unavailable imaging at the treatment completion timepoint (*n* = 5), interval surgery during treatment (*n* = 4), missing images (*n* = 8) and failure of registration of ADC map to post-contrast T1-weighted imaging (*n* = 1). Of the 56 patients who met the inclusion criteria, 18 patients were excluded. A diagram of the patient selection process is shown in Figure 1.

### 2.2. MRI Acquisition and Post Processing

Images were obtained on 1.5 T and 3T MRI scanners at the two treatment centres and their referring institutions. MRI studies included DWI series and T1-weighted series with gadolinium-based contrast agent. Either 0.1 mmol/kg DOTAREM (Guerbet, Villepinte, France) or 0.1 mmol/kg ProHance (Bracco, Milan, Italy) contrast agent was used.

Images were acquired on 14 different MRI scanners (GE MR450, GE MR750w, GE SIGNA EXCITE, GE Signa HDxt, Philips Ingenia, Philips Achieva, Philips Ingenia, Philips Intera, Siemens Aera, Siemens Avanto, Siemens Espree, Siemens Prisma_fit, Siemens Skyra, Siemens SymphonyTim) at either 1.5 or 3 T. The range (min-max) of image acquisition parameters were as follows, and listed in full for each DWI examination in Table 2 and Table 3: T1w (TE 1.7–18 ms, TR 5–2200 ms, slice thickness 1–5 mm, slice gap (mm) 0.5–6.5 mm, in-plane voxel size 0.4–1.0 mm) and DWI (b-values 0 and 1000 s/mm^2^) TE 55–115 ms, TR 2660–12,025 ms, slice thickness 2.5–5 mm, slice gap (mm) 0–0.5 mm, in-plane voxel size 0.6–2.5 mm). Both gradient echo and fast spin echo based sequences were used at different sites to acquire the T1-weighted images.

T1CE tumour volumes of interest were outlined at baseline, after 2 cycles of MATRix and after 4 cycles of MATRix using ITK SNAP Toolbox Version 3.6.0 [18] (www.itksnap.org; Philadelphia, PA, USA), by a neuroimaging student (IC) blinded to IPCG response assessments, covering the entire post-contrast T1 signal abnormality on axial views. All segmentations were supervised and, where necessary, optimised by a neuroradiologist specialised in oncology (ST, 11 years’ experience). Post-treatment scans following 2 cycles and 4 cycles of chemotherapy were segmented side by side, comparing pre- and post-contrast images in order to avoid segmentation of non-enhancing T1 hyperintense tissue (T1 shortening). T1CE tumour volumes (cm^3^) were calculated and their mean and standard deviation parameters generated. The absolute change in tumour volume was calculated (=volume after 2 cycles of MATRix–baseline volume) as well as the percentage (%) change in tumour volume (=change in tumour volume/baseline volume in %).

ADC maps were calculated from 3-directional DWI acquired with 2 gradient values (b0 and b1000 s/mm^2^) using proprietary software (Olea Sphere, version 2.3, Olea Medical, La Ciotat, France). In the generation of an ADC map, the image acquired without diffusion gradients is divided by the image acquired with diffusion gradients and the natural logarithm is taken, removing dependence on T1, T2 and TR [19]. Sufficient comparability of ADC between scanners has been demonstrated previously [20]. Registration to ADC maps was performed using the FSL FLIRT toolbox (Linear Image Registration Tool, used for inter- and intra-modal registration [21,22]. A rigid transformation with either a 12 or 6 parameter model and Normalized Mutual Information as the cost function. The quality of the image registration was assessed visually by a neuroradiologist. In cases where the 6-parameter model did not result in a good spatial alignment, a 12-parameter model was used. This resulted in good alignment in all cases. Through this process, ADC of the tumour corresponding to the entire T1CE lesion was generated. We extracted the ADC value of every voxel within the ROI. Subsequently, ADC histogram data were obtained for each tumour region of interest, using an in-house script written in Python 2.7. We then calculated statistics from this histogram for each tumour, including the 2nd, 25th, 50th and 75th ADC percentiles. From the measurable histogram data, the mean ADC value (ADC_mean_) and cumulative parameters including the 2nd (ADC_min_) and 25th (ADC_2_5th) percentiles prior to treatment (‘baseline’) and after 2 cycles of treatment (‘early’) were used. Our hypothesis was that we would see reduced ADC at baseline in non-responses so we evaluated the minimum and lower percentiles of this histogram. Consistent with recommendations from other MRI analysis software packages such as FSL, we noticed that the raw ADC_min_ was susceptible to outliers so used the 2nd centile ADC as a proxy of the minimum. From here on, ADC_min_ = 2nd percentile ADC. Using these values change in ADC (ADC_change_ = ADC after 2 cycles of MATRix–ADC at baseline) and percentage ADC change (= ADC_change_/baseline ADC) were calculated. ADC values are reported as 100 × 10^−6^ mm^2^/s.

### 2.3. Consensus Response Evaluation

All imaging was analysed in an anonymised format, blinded to tumour volumes and ADC measurements, by two board-certified neuroradiologists (AAB with 6 years of experience and ST with 11 years of experience in MR brain imaging). By consensus, categorical outcomes were specified according to the radiological criteria as defined by the International PNCSL Collaborative Group. Outcome was assessed at two time points as follows: early during treatment (after 2 cycles of MATRix) and on completion of chemotherapy (after 4 cycles of MATRix). All imaging studies following completion of treatment were carried out within one month of chemotherapy cessation.

**Figure 2:**
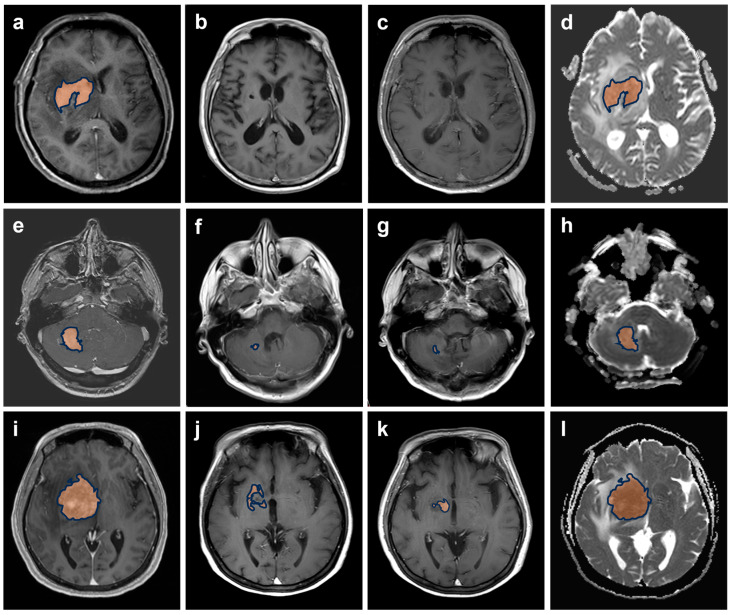
An example of the response groups defined for statistical analysis. T1CE-weighted images with manually segmented tumours before treatment (first column) and after completion of 2 and 4 cycles of MATRix immunotherapy (second and third columns, respectively) in a patient with complete response (Group 0, (**a**–**c**)), a patient with partial response (Group 1, (**e**–**g**)) and a patient with lack of response and with appearance of a new lymphoma deposit from 2 to 4 cycles of treatment (Group 2, (**i**–**k**)). Corresponding ADC images at baseline are shown for each example (Group 0, (**d**); Group 1, (**h**); Group 2, (**l**)).

To facilitate statistical analysis and to be consistent with prior research [4,23,24], radiological outcomes were grouped into three response categories. All complete response (CR, no residual lesion enhancement) and unconfirmed complete response (CRu, minimal enhancement) patients formed the ”complete response” category (Group 0). The next group comprised all patients who showed a partial response (Group 1), defined as >50% reduction in the size of enhancing lesion(s), but not meeting criteria for CR/CRu. Group 2 comprised patients who lacked response, either in the form of stable disease (SD) or progressive disease (PD). Example images for the three different response groups are shown in Figure 2.

### 2.4. Statistical Analysis

All analyses were performed using Statistical Package for the Social Sciences version 25 (IBM SPSS 25, Chicago, IL, USA). Using the Wilcoxon non-parametric test, multiple comparison analysis was performed to identify whether the response assessment after 2 cycles of MATRix could qualitatively predict the response assessment after completion of MATRix.

One-way analysis of variance (ANOVA) with post hoc analysis using the least significant difference method was carried out to confirm whether differences seen between groups are unlikely due to random chance. For this, we evaluated the tumour volume at baseline, absolute and percentage change in tumour volume, baseline ADC_min_ (2nd centile), baseline ADC_mean_, baseline ADC_2_5th, ADC_min_ change, ADC_mean_ change and ADC_2_5th change. Each of the (continuous variable) quantitative metrics were statistically tested against the three outcomes (0 = CR/CRu, 1 = PR, 2 = SD/PD) following completion of treatment.

Univariate multinomial logistic regression was then used to test the tumour volume and ADC parameters as predictors of response assessment after completion of treatment. Significant (*p* < 0.05) predictors identified by univariate analysis were then combined and tested in a multivariate logistic regression model for prediction of response. A two-sided *p* value was used with results of *p* < 0.05 considered statistically significant.

## 3. Results

### 3.1. Overview

Thirty-eight patients met criteria for inclusion in the study. By the IPCG standard assessment, 20 patients were classified as “Group 0” (complete response), 12 patients as “Group 1” (partial response or stable disease) and 6 patients as “Group 2” (non-responders).

### 3.2. Predicting End of Treatment Response by T1CE Baseline Tumour Volume

**Figure 3:**
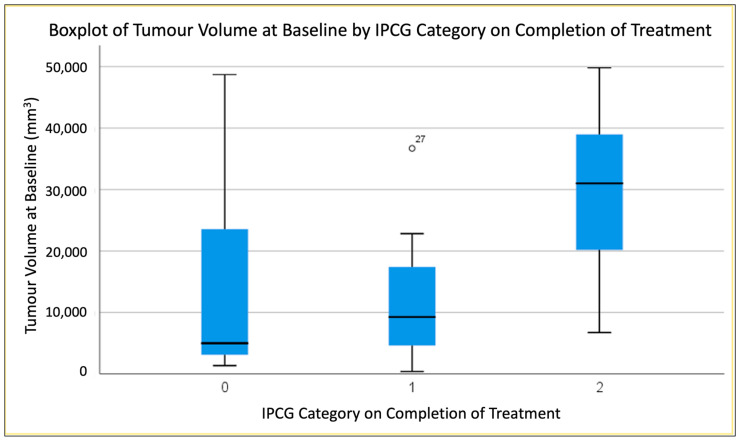
Boxplot demonstrating difference in Baseline Volumes between treatment response groups.

T1CE tumour volume at baseline significantly differed amongst response groups 0, 1 and 2 (Figure 3). ANOVA testing showed that Group 2 had statistically significant larger tumour volumes at baseline compared to Group 0 (*p* < 0.020) and compared to Group 1 (*p* < 0.014). There was no significant difference in baseline tumour volume between Group 0 and Group 1. In one patient, T1 shortening due to macroscopic haemorrhage was present at baseline, but no pre-contrast axial imaging was available for segmentation. Therefore, both pre- and post-contrast segmentations were performed on coronal T1 sequences to optimise volume of interest placements. The pre-contrast segmentations were then subtracted from the post-contrast segmentations to remove haemorrhage from the tumour, enhancing volume calculation.

### 3.3. Predicting End of Treatment Response by T1CE Percentage Volume Change (Baseline to 2 MATRix Cycles)

There was a borderline significant difference in change in absolute tumour volume from baseline to the completion of two treatment cycles between Group 1 and Group 2, with a smaller reduction in T1CE volume in non-responders (*p* < 0.047). Percentage (%) change in T1CE volume differed between the groups: There was a greater % reduction in tumour volume in Group 0 compared to Group 1 (*p* < 0.018) and to Group 2 (*p* < 0.011). No significant difference in % change in T1CE tumour volume was found between groups 1 and 2 (*p* < 0.499).

### 3.4. Predicting End of Treatment Response by ADC Values at Baseline

ADC values at baseline varied across groups, from largest values for the complete response group to smallest values for the progressive disease group.

#### 3.4.1. Second-Percentile ADC (ADC2nd) at Baseline

**Figure 4:**
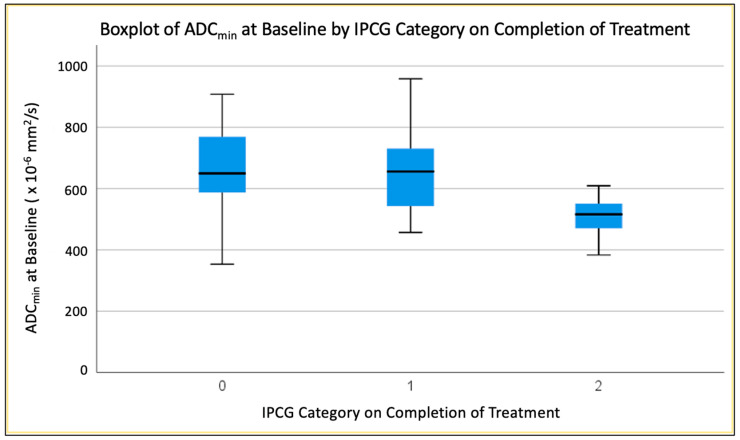
Boxplot demonstrating difference in Baseline ADC_min_ between groups.

There was a significant difference in second-percentile ADC at baseline between groups 0 and 2 (*p* < 0.013) and between groups 1 and 2 (*p* < 0.031), with lower ADC values corresponding to less response (Figure 4). No significant difference in second-percentile ADC at baseline was evident between complete response (0) and partial (1) response groups (*p* < 0.410).

#### 3.4.2. Twenty-Fifth-Percentile ADC (ADC_2_5th) at Baseline

There was a significant difference in this parameter between the complete response (0) and progressive disease (2) groups (*p* < 0.011) and also between partial response (1) and complete response groups (0) (*p* < 0.0032) with lower ADC values corresponding to less response. There was no significant difference between complete response and partial response groups (*p* < 0.719).

#### 3.4.3. Mean ADC (ADC_mean_) at Baseline

**Figure 5:**
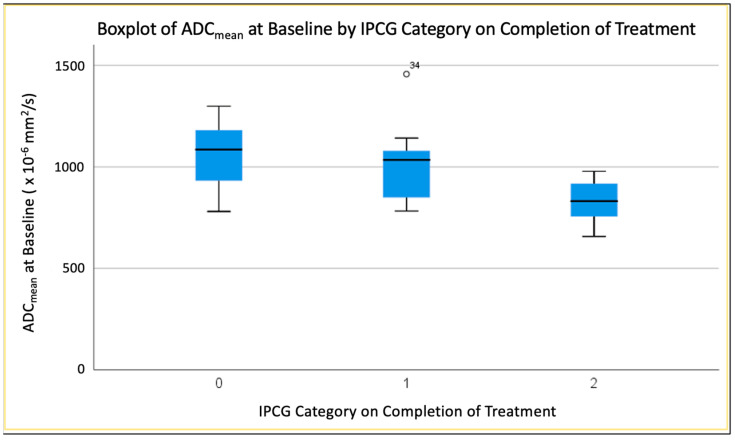
Boxplot demonstrating difference in Baseline ADC_mean_ between groups.

The most strongly significant difference was in ADC_mean_ at baseline between complete response (0) and progressive disease (2) groups (*p* < 0.005) (Figure 5). There was also a significant difference in ADC_mean_ at baseline between partial response (1) and progressive disease (2) groups (*p* < 0.038). There was no significant difference in this parameter between complete response and partial response groups (*p* < 0.410).

### 3.5. Radiologist Early (after 2 Cycles) Compared to Later (after 4 Cycles) Assessment by IPCG Criteria

Response by IPCG criteria after two cycles of MATRiX chemotherapy differed significantly from response after four cycles (Wilcoxon *p* = 0.023), whereby 33% patients (*n* = 2) with progressive disease on MATRix therapy completion had initially shown complete or partial response after two cycles.

### 3.6. Logistic Regression

Univariate multinomial logistic regression to test T1CE tumour volume at baseline as a biomarker of response showed moderately good IPCG category prediction at the end of four cycles’ treatment (*p* < 0.042) but did not predict response after two cycles of treatment (*p* < 0.647). The area under the Receiver Operator Characteristics (ROC) curve (AUC) for prediction at 4 weeks was 0.828.

Regression to test % change in T1CE tumour volume showed that this parameter is a predictor of outcome after two cycles of treatment (*p* = 0.025) and after four cycles of treatment (*p* = 0.016). However, the AUC for this parameter was lower (0.672) than using baseline T1 volume for predictions.

**Figure 6:**
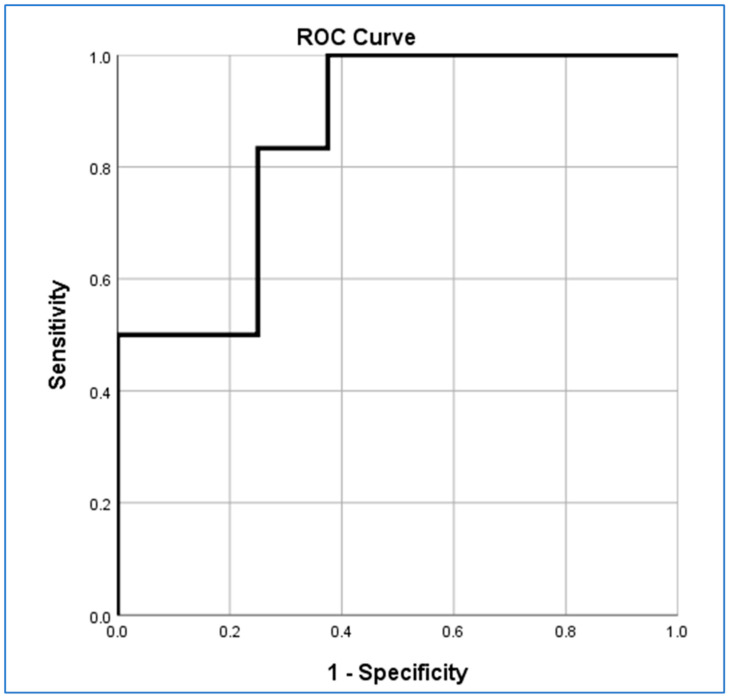
Receiver Operator Characteristic curve for ADC_mean_ at baseline.

ADC_mean_ at baseline was the strongest predictor of IPCG response at the end of four cycles’ treatment (*p* < 0.017, AUC 0.854; Figure 6) amongst the parameters tested but, interestingly, this did not predict response after two cycles of treatment (*p* < 0.414).

There was no accuracy improvement derived from combining the individual predictive parameters in a multivariate logistic regression model for prediction of IPCG-categorised radiological response.

## 4. Discussion

In this study, we aimed to identify quantifiable biomarkers predictive of PCNSL response after completion of MATRix chemotherapy. The research was undertaken using clinical MRI data from two large UK centres with specialist lymphoma services.

Contrast-enhancing tumour percentage change estimation represents the international standard by which PCNSL response is recorded using semi-quantitative IPCG criteria [1]. The limitations of two-dimensional response approximation in brain tumours have been widely acknowledged [25]. Moreover, the value of serial tumour volumetry has been recognised, for example, in gliomas [26,27], and is deemed to be more accurate particularly for non-spherical lesions, and the latest IPCG guidelines recommend volumetric T1CE in all patients with (suspected) PCNSL [10].

In our dual-centre patient cohort, we observed that PCNSL baseline T1CE lesion volumes differ between IPCG response groups with larger tumour volumes observed in individuals non-responsive to MATRix after the completion of four cycles (*p* < 0.02). This finding can be explained by the more extensive intracranial disease load, which may exacerbate limitations to chemo agent penetration through the blood–brain barrier [28]. The group differences appeared greater when assessing baseline tumour volumes than when calculating dynamic % changes in tumour volumes between baseline and end of cycle 2 (weakly significant *p* < 0.047).

A recent study using T1-weighted dynamic contrast-enhanced perfusion MRI identified higher values of permeability metrics (Ktrans, Ve) predictive of poor response when measured at baseline and when assessing change early during combination chemotherapy for PCNSL [29]. Despite the MRI sequence differences, it is noteworthy that for both modalities the result trends are similar, with greater T1-weighted abnormalities indicating non-response. No further results on T1CE-based PCNSL response prediction were identified in a dedicated literature search.

In this study, baseline T1CE total tumour volume predicted PCNSL response as assessed by IPCG criteria upon completion of four MATRix cycles (*p* < 0.042, AUC 0.828). This observation could be valuable in identifying patients prone to suboptimal therapy outcome. Furthermore, differential response data could potentially inform the design of future multicentre PCNSL trials to test new treatment strategies (https://clinicaltrials.gov/ct2/show/NCT049313680, accessed on 15 May 2023). Percentage change in T1CE tumour volume predicted MATRix response at 2 weeks (*p* < 0.025) and following completion of treatment (*p* < 0.016); however, this result was less exact (0.672) compared to baseline volume-derived outcome prediction.

Low ADC_min_, ADC_mean_ and ADC_max_ values have been shown to reflect a high cellularity and proliferative activity in PCNSL patients [30]. Our research identified differences in baseline diffusivity between MATRix response groups, particularly in ADC_mean_ between complete responders and non-responders (*p* < 0.005) and between partial response (1) and progressive disease (2) groups (*p* < 0.038). ADC_mean_ at baseline seemed marginally superior to T1CE baseline tumour volume as the most accurate predictor of IPCG response (*p* < 0.017, AUC 0.854). The value of ADC measurements for the characterisation of PCNSL has been highlighted numerous times, most commonly in the context of differential diagnosis [31,32,33], and ADC has long been recognised as potentially valuable for brain tumour response assessment generally [34,35].

The first study to assess ADC values in association to outcome in (methotrexate-only) chemotherapy for PCNSL was by Barajas et al. [23]. In this research, all (*n* = 18) patients in the high-ADC group at baseline showed complete remission. Baseline minimum ADC (*p* < 0.01), 25th-percentile ADC (*p* < 0.01) and mean ADC (*p* = 0.02) values were significantly lower for seven patients who showed only partial response or progressive disease after completion of treatment. Similar to our results, Barajas proposed that baseline ADC values could predict clinical response following induction chemotherapy. Subsequently, Wieduwilt and colleagues [24] measured pre-treatment minimum ADC for a correlate to survival in patients (*n* = 23) undergoing induction with methotrexate, temozolomide and rituximab followed by consolidation with etoposide and cytarabine. In this study, patients in the low-ADC group had a shorter median progression-free survival (PFS) (*p* = 0.007), and restricted diffusion (defined as minimum ADC < 384 × 10^−6^ mm^2^/s) signified shorter OS (*p* = 0.003). The authors suggested that tumour ADC values were a better prognostic factor than clinical data (e.g., performance scores).

Similar to our results, a study by Valles et al. with 25 patients identified low ADC values as predictive of unfavourable outcome when analysing PCNSL response by survival [36]. In this study, patients with minimum ADC values < 384 × 10^−6^ mm^2^/s had worse PFS and overall survival (OS). Contrary to our data, Valles identified no relationship between contrast-enhancing lesion size and PFS or OS. The therapeutic approach in this study also slightly differed to ours with patients receiving a combination of methotrexate, temozolomide and rituximab therapy for PCNSL. Our analysis did not assess image parameters for prediction of survival, and it is noteworthy that response defined by IPCG criteria may not necessarily be an accurate measure of long-term patient outcome [9]. In a later study, Huang et al. examined pre-treatment tumour diameter for prediction of chemotherapy response (methotrexate and idarubicin, *n* = 35) [4]. The authors of this study found that pre-treatment minimum ADC in the progressive disease group was lower than that in the complete response and partial response groups. Huang et al. also observed that minimum ADC after one cycle and minimum ADC changes were better correlated with the treatment response than the pre-treatment minimum ADC, which slightly differs from our results.

In a different cohort of PCNSL patients receiving methotrexate-only chemotherapy (n = 28), Zhang et al. assessed the ability of baseline ADC parameters to distinguish between patients with complete and partial response [11]. Fifth-percentile and mean ADC values significantly differed response groups after four methotrexate cycles, with fifth-percentile (AUC 0.983) superior to mean ADC values (0.822).

The combination of study data, despite some variations in treatment regimen and parameters analysed, suggests that ADC metrics are likely to be a valuable biomarker predictive of outcome in PCNSL. To the authors’ knowledge, no previous research has assessed clinical imaging parameters in patients receiving MATRix chemotherapy, a treatment regimen which became standard in 2016 [6]. Therefore, the literature context includes studies assessing MRI parameters in patients receiving methotrexate therapy, either alone or in combination with other chemotherapy agents (rituximab, temozolomide or idarubicin) as described. Given the toxicity of these treatment regimens, including early (mostly infectious) complications which occur in up to 28% of patients [7] and diminished therapeutic success, early predictions of response may reduce overtreatment and/or side effects.

Limitations of the previous studies and ours are small patient numbers with PCNSL being a rare disease. As a multi-center clinical study, there was also heterogeneity in image acquisition, with studies acquired on both 3T and 1.5T MRI systems with different parameters. It has been shown that diffusion MRI parameters for clinical sequences in the brain, including ADC, are robust across different scanner systems, including those of different field strengths [20]. In addition, for texture analysis of MRI of brain tumours, there is a real risk of overfitting when using limited and uniform image data, and multi-center and multi-vendor image data is now encouraged [37].

A further limitation of this study arises from the imperfection of the IPCG reference standard, which may not consistently translate into survival differences. However, because assessment by IPCG criteria represents the basis for PCNSL trial decisions worldwide, it was deemed the most suitable short-term outcome reference.

Manual tumour segmentation is time-consuming and can be limited by intra- and inter-rater variabilities, also a potential limitation in our study. That said, in a previous study by our group on patients with WHO grade 2/3 gliomas, we found high reproducibility of region of interest ADC parameters and contrast-enhancement patterns among 3 independent raters (intraclass correlation coefficient = 0.83–0.96 and Cohen k = 0.69–0.72, respectively) [38]. We note similar methodologies have been applied in comparable studies (for example [39]) and it has also been shown in head and neck squamous cell carcinoma that ADC values can be reproducibly obtained by different radiologists pre- and post-chemoradiotherapy [40]. Nevertheless, development of automated software tools based on deep learning may improve the consistency of tumour delineation. Promisingly, a 3-dimensional convolutional neural network trained on gliomas has been found to be able to provide automatic segmentations of PCNSL tumours comparable to manual segmentation [41].

This study makes an important knowledge contribution through identification of possible T1CE and ADC biomarkers, which may predict response to MATRix at baseline. Longer-term follow up is planned to clarify in how far the candidate biomarkers relate to overall survival. Computational analysis of image data [31,42] could be explored as an adjunct for image-based outcome predictions though this would ideally benefit from large data sets for training.

## 5. Conclusions

The data from this study indicate a potential role for volumetric T1CE and ADC measurements as biomarkers predictive of response to MATRix chemotherapy, a recently adopted standard-of-care regimen for newly diagnosed PCNSL. Contrast-enhanced and diffusion parameters appeared similarly accurate when assessed at baseline. This research was conducted using widely adopted MRI brain protocols with a method that could be suitable for clinical translation.

## Figures and Tables

**Table 1:** Response Assessment on Imaging as per IPCG recommendations.

Response	Brain Imaging
CR	Disappearance of all enhancing abnormalities on gadolinium-enhancing MRI
CRu	Small but persistent abnormality on MRI related to biopsy or focal haemorrhage, found to represent scar tissue on serial scans
PR	≥50% decrease in contrast-enhancing lesion seen on MRI as compared with baseline imaging
PD	>25% increase in contrast-enhancing lesion on MRI compared with baseline or best response

**Table 2:** DWI acquisition parameters for patients imaged at or referred to NHNN. All patients were scanned with b-values of 0 and 1000 s/mm^2^.

Subject	Manufacturer	Model	Field Strength (T)	Series	TE (ms)	TR (ms)	Slice Thickness (mm)	Slice Spacing (mm)	In Plane Resolution (mm × mm)
NHNN_001a	Siemens	SymphonyTim	1.5	00005-MR-ep2d_diff_3scan_trace_p2	106	4400	5	6.5	1.2 × 1.2
NHNN_001b	Siemens	Avanto	1.5	00007-MR-DWI_Tra	81	3734	5	6.5	1.8 × 1.8
NHNN_002a	Siemens	Avanto	1.5	00004-MR-ep2d_diff_3scan_trace_p2	102	3723	5	6.5	1.2 × 1.2
NHNN_002b	Siemens	Avanto	1.5	00007-MR-DWI_Tra	81	3200	5	6.5	1.8 × 1.8
NHNN_003a	Siemens	Espree	1.5	00007-MR-ep2d_diff_3scan_trace_aa	100	4000	5	6.5	1.6 × 1.6
NHNN_003b	Siemens	SymphonyTim	1.5	00007-MR-DWI_Tra	84	3200	5	6.5	1.8 × 1.8
NHNN_003c	Siemens	Avanto	1.5	00007-MR-DWI_Tra	81	3348	5	6.5	1.8 × 1.8
NHNN_004a	Siemens	Avanto	1.5	00006-MR-ep2d_diff_3scan_trace	102	4300	5	6.5	1.2 × 1.2
NHNN_004b	Siemens	Avanto	1.5	00007-MR-DWI_Tra	81	3200	5	6.5	1.8 × 1.8
NHNN_004c	Philips	Achieva	3	00401-MR-dwi_1000	95	2900	5	6	0.9 × 0.9
NHNN_005b	Siemens	SymphonyTim	1.5	00009-MR-DWI_Tra	84	3200	5	6.5	1.8 × 1.8
NHNN_005c	Philips	Achieva	3	00401-MR-dwi_1000	96	2934	5	6	0.9 × 0.9
NHNN_006a	Philips	Achieva	3	00401-MR-dwi_1000	96	2934	5	6	0.9 × 0.9
NHNN_006b	Siemens	Avanto	1.5	00007-MR-DWI_Tra	81	3348	5	6.5	2.0 × 2.0
NHNN_007a	Siemens	Avanto	1.5	00005-MR-ep2d_diff_3scan_trace_p2_aa	89	3600	5	6.5	1.3 × 1.3
NHNN_008a	Philips	Achieva	1.5	00501-MR-sDW_SSh	90	3174	5	6	1.8 × 1.8
NHNN_009a	GE Medical Systems	SIGNA Excite	1.5	00004-MR-Ax_DWI_1000b	100	9000	4	4.4	1.2 × 1.2
NHNN_009b	Siemens	Avanto	1.5	00007-MR-DWI_Tra	81	3200	5	6.5	1.8 × 1.8
NHNN_010a	GE Medical Systems	Discovery MR450	1.5	00008-MR-Ax_DWI_(NEW)	83	8000	5	6	0.9 × 0.9
NHNN_010b	Siemens	Avanto	1.5	00008-MR-DWI_Tra	81	3200	5	6.5	1.8 × 1.8
NHNN_011a	Siemens	Avanto	1.5	00010-MR-ep2d_diff_3scan_trace_p2_aa	89	3600	5	6.5	1.3 × 1.3
NHNN_011b	Siemens	Avanto	1.5	00005-MR-DWI_Tra	81	3200	5	6.5	1.8 × 1.8
NHNN_012a	Siemens	Espree	1.5	00005-MR-DWI_3scanTrace_2.5iso	87	6800	2.5	2.5	2.5 × 2.5
NHNN_012b	Siemens	Avanto	1.5	00008-MR-ep2d_diff_3scan	81	3200	5	6.5	1.8 × 1.8
NHNN_012c	Siemens	Avanto	1.5	00007-MR-DWI_Tra	76	3200	5	6.5	1.8 × 1.8
NHNN_013a	Siemens	Prisma_fit	3	00007-MR-resolve_4scan_trace_ p2_192_TRACEW	55	3700	5	6.5	1.1 × 1.1
NHNN_013b	Philips	Achieva	3	00401-MR-dwi_1000	96	2933	5	6	0.9 × 0.9
NHNN_013c	Philips	Ingenia	3	00702-MR-Reg_-_DWI_3b_Tra_SENSE	99	3961	5	5.5	1.0 × 1.0
NHNN_014a	Siemens	Avanto	1.5	00005-MR-ep2d_diff_3scan_trace_p2	102	3400	5	6.5	1.2 × 1.2
NHNN_014b	Philips	Achieva	3	00401-MR-dwi_1000	96	2933	5	6	0.9 × 0.9
NHNN_015a	Siemens	Skyra	3	00009-MR-ep2d_diff_3scan_trace_p2_aa_TRACEW	98	6000	5	6.5	1.1 × 1.1
NHNN_015b	Siemens	Avanto	1.5	00009-MR-DWI_Tra	81	3900	5	6.5	1.8 × 1.8
NHNN_016a	Siemens	Avanto	1.5	00010-MR-ep2d_diff_3scan_trace_p2_aa	89	3600	5	6.5	1.3 × 1.3
NHNN_016b	Siemens	SymphonyTim	1.5	00007-MR-DWI_Tra	84	3200	5	6.5	1.8 × 1.8
NHNN_016c	Philips	Ingenia	3	00801-MR-DWI_3b_ax_cc	101	4392	5	5.5	1.0 × 1.0
NHNN_017a	GE Medical Systems	Signa HDxt	1.5	00004-MR-Ax_DWI_1000b	82	8000	4	4.5	1.1 × 1.1
NHNN_017b	GE Medical Systems	Signa HDxt	1.5	00004-MR-Ax_DWI_1000b	82	8650	4	4.5	1.1 × 1.1
NHNN_018a	Siemens	Avanto	1.5	00004-MR-ep2d_diff_3scan_trace_p2_aa	89	3600	5	6.5	1.3 × 1.3
NHNN_020a	Siemens	Prisma_fit	3	00007-MR-resolve_4scan_trace_ p2_192_TRACEW	55	3700	4	5	1.1 × 1.1
NHNN_020b	Philips	Ingenia	3	00801-MR-DWI_3b_ax_cc	99	3917	5	5.5	1.0 × 1.0
NHNN_020c	Siemens	Avanto	1.5	00008-MR-DWI_Tra	81	3200	5	6.5	1.8 × 1.8
NHNN_021a	Siemens	Avanto	1.5	00007-MR-ep2d_diff_3scan_trace_p2	102	3200	5	6.5	1.2 × 1.2
NHNN_021b	Philips	Achieva	3	00501-MR-dwi_1000	95	2939	5	6	0.9 × 0.9
NHNN_022b	Siemens	Avanto	1.5	00007-MR-DWI_Tra	81	3200	5	6.5	1.8 × 1.8
NHNN_023a	Siemens	Skyra	3	00012-MR-ep2d_diff_3scan_trace_p2_aa_TRACEW	98	6000	5	6.5	1.1 × 1.1
NHNN_024a	Siemens	Avanto	1.5	00005-MR-ep2d_diff_3scan_trace_p2_aa	89	3600	5	6.5	1.3 × 1.3
NHNN_025a	Siemens	Avanto	1.5	00006-MR-ep2d_diff_3scan_trace_p2	102	4100	5	6.5	1.2 × 1.2
NHNN_026b	Siemens	Avanto	1.5	00007-MR-DWI_Tra	81	3200	5	6.5	1.8 × 1.8
NHNN_026c	Siemens	SymphonyTim	1.5	00010-MR-DWI_Tra	84	3200	5	6.5	1.8 × 1.8
NHNN_028a	Siemens	Avanto	1.5	00011-MR-DWI_Tra	81	3200	5	6.5	1.8 × 1.8

**Table 3:** DWI acquisition parameters for patients imaged at or referred to NUH. All patients were scanned with b-values of 0 and 1000 s/mm^2^.

Subject	Manufacturer	Model	Field Strength (T)	Series	TE (ms)	TR (ms)	Slice Thickness (mm)	Slice Spacing (mm)	In Plane Resolution (mm × mm)
Nott_020a	GE Medical Systems	Signa HDxt	1.5	00004-MR-DWI	81	8000	4	5	1.0 × 1.0
Nott_026b	GE Medical Systems	Signa HDxt	1.5	00004-MR-DWI	81	8000	4	5	1.0 × 1.0
Nott_060a	Philips	Achieva	3	00401-MR-DW_SSh_new_2012	55	2661	4	5	1.0 × 1.0
Nott_060b	GE Medical Systems	Signa HDxt	1.5	00004-MR-DWI	82	7000	4	5	1.0 × 1.0
Nott_063a	GE Medical Systems	Signa HDxt	1.5	00004-MR-Ax_DWI	82	8000	4	5	0.9 × 0.9
Nott_063b	GE Medical Systems	Signa HDxt	1.5	00004-MR-DWI	82	7000	4	5	1.0 × 1.0
Nott_063c	GE Medical Systems	Signa HDxt	1.5	00004-MR-DWI	82	7000	4	5	1.0 × 1.0
Nott_066a	Siemens	Avanto	1.5	00006-MR-ep2d_diff_3scan_trace_p2	102	4100	5	6.5	1.2 × 1.2
Nott_066b	GE Medical Systems	Signa HDxt	1.5	00004-MR-DWI	81	7175	4	5	1.0 × 1.0
Nott_066c	Siemens	Aera	1.5	00007-MR-ep2d_diff_3scan_trace_p2_TRACEW	89	6300	5	6.5	0.6 × 0.6
Nott_067a	GE Medical Systems	Signa HDxt	1.5	00004-MR-DWI	81	7000	4	5	1.0 × 1.0
Nott_067b	Siemens	Aera	1.5	00007-MR-resolve_4scan_trace_tra_160_p2_TRACEW	60	6150	4	4.96	1.4 × 1.4
Nott_068b	Siemens	Aera	1.5	00007-MR-resolve_4scan_trace_tra_160_p2_TRACEW	60	6330	4	4.96	1.6 × 1.6
Nott_068c	Philips	Achieva	3	00601-MR-DWI	96	4176	4	4.4	0.9 × 0.9
Nott_070a	Philips	Achieva	3	00601-MR-DWI	96	4043	4	4.4	0.9 × 0.9
Nott_070b	Siemens	Aera	1.5	00006-MR-resolve_4scan_trace_tra_160_p2_DWI_TRACEW	60	6330	4	4.96	1.6 × 1.6
Nott_070c	Siemens	Aera	1.5	00006-MR-resolve_4scan_trace_tra_160_p2_DWI_TRACEW	60	6330	4	4.96	1.6 × 1.6
Nott_072a	GE Medical Systems	Signa HDxt	1.5	00006-MR-Ax_DWI	82	8000	4	5	0.9 × 0.9
Nott_072b	Siemens	Aera	1.5	00006-MR-resolve_4scan_trace_tra_160_p2_DWI_TRACEW	60	6560	4	4.96	1.6 × 1.6
Nott_077a	Philips	Achieva	3	00601-MR-DWI	95	4008	4	4.4	0.9 × 0.9
Nott_077b	Siemens	Aera	1.5	00006-MR-resolve_4scan_trace_tra_160_p2_DWI_TRACEW	60	6330	4	4.96	1.6 × 1.6
Nott_077c	Siemens	Aera	1.5	00006-MR-resolve_4scan_trace_tra_160_p2_DWI_TRACEW	60	6330	4	4.96	1.6 × 1.6
Nott_080a	GE Medical Systems	Signa HDxt	1.5	00003-MR-Ax_DWI	82	8000	4	5	0.9 × 0.9
Nott_080b	Siemens	Aera	1.5	00007-MR-resolve_4scan_trace_tra_160_p2_DWI_TRACEW	60	6330	4	4.96	1.6 × 1.6
Nott_080c	Siemens	Aera	1.5	00005-MR-ep2d_diff_3scan_trace_p2_TRACEW	89	8200	4	5	0.6 × 0.6
Nott_081a	Philips	Achieva	1.5	00501-MR-DWI	89	4119	4	5	1.0 × 1.0
Nott_081b	Siemens	Aera	1.5	00005-MR-ep2d_diff_3scan_trace_p2_TRACEW	89	8200	4	5	0.6 × 0.6
Nott_082a	GE Medical Systems	Signa HDxt	1.5	00007-MR-Ax_DWI	82	8000	4	5	0.9 × 0.9
Nott_082b	Siemens	Aera	1.5	00005-MR-ep2d_diff_3scan_trace_p2_TRACEW	89	8200	4	5	0.6 × 0.6
Nott_082c	Siemens	Aera	1.5	00005-MR-ep2d_diff_3scan_trace_p2_TRACEW	89	8800	4	5	0.6 × 0.6
Nott_083a	Siemens	Aera	1.5	00005-MR-ep2d_diff_3scan_trace_p2_TRACEW	89	8200	4	5	0.6 × 0.6
Nott_083b	Philips	Achieva	3	00601-MR-DWI	95	4007	4	4.4	0.9 × 0.9
Nott_085a	Siemens	Aera	1.5	00005-MR-ep2d_diff_3scan_trace_p2_TRACEW	89	8800	4	5	0.6 × 0.6
Nott_085b	Siemens	Aera	1.5	00005-MR-ep2d_diff_3scan_trace_p2_TRACEW	89	9000	4	5	0.6 × 0.6
Nott_085c	Siemens	Aera	1.5	00006-MR-resolve_4scan_trace_tra_160_p2_DWI_TRACEW	60	6780	4	4.96	1.6 × 1.6
Nott_086a	Philips	Achieva	3	00601-MR-DWI	95	4541	4	4.4	0.9 × 0.9
Nott_086b	Siemens	Aera	1.5	00007-MR-resolve_4scan_trace_tra_160_p2_DWI_TRACEW	60	6780	4	4.96	1.6 × 1.6
Nott_086c	Siemens	Aera	1.5	00010-MR-resolve_4scan_trace_tra_160_p2_DWI_TRACEW	60	7010	4	4.96	1.6 × 1.6
Nott_087a	Philips	Intera	1.5	00601-MR-sDW_SSh	91	4727	4	5	2.0 × 2.0
Nott_087b	Siemens	Aera	1.5	00005-MR-ep2d_diff_3scan_trace_p2_TRACEW	89	8200	4	5	0.6 × 0.6
Nott_087c	Siemens	Aera	1.5	00006-MR-resolve_4scan_trace_tra_160_p2_DWI_TRACEW	60	6560	4	4.96	1.6 × 1.6
Nott_095a	Siemens	Aera	1.5	00005-MR-ep2d_diff_3scan_trace_p2_TRACEW	115	6700	4	4.4	1.3 × 1.3
Nott_095b	Siemens	Aera	1.5	00005-MR-ep2d_diff_3scan_trace_p2_TRACEW	89	8200	4	5	0.6 × 0.6
Nott_095c	Siemens	Aera	1.5	00005-MR-ep2d_diff_3scan_trace_p2_TRACEW	89	8200	4	5	0.6 × 0.6
Nott_099a	Philips	Achieva	3	00401-MR-DWI	96	4077	4	4.4	0.9 × 0.9
Nott_099b	Siemens	Aera	1.5	00006-MR-resolve_4scan_trace_tra_160_p2_DWI_TRACEW	60	6780	4	4.96	1.6 × 1.6
Nott_099c	Siemens	Aera	1.5	00006-MR-resolve_4scan_trace_tra_160_p2_DWI_TRACEW	60	6330	4	4.96	1.6 × 1.6
Nott_100a	Siemens	Aera	1.5	00005-MR-ep2d_diff_3scan_trace_p2_TRACEW	89	8200	4	5	0.6 × 0.6
Nott_100b	Siemens	Aera	1.5	00005-MR-ep2d_diff_3scan_trace_p2_TRACEW	89	8200	4	5	0.6 × 0.6
Nott_100c	Philips	Achieva	3	00601-MR-DWI	95	4044	4	4.4	0.9 × 0.9
Nott_103a	Siemens	Aera	1.5	00005-MR-ep2d_diff_3scan_trace_p2_TRACEW	89	8200	4	5	0.6 × 0.6
Nott_103b	Siemens	Aera	1.5	00005-MR-ep2d_diff_3scan_trace_p2_TRACEW	89	8200	4	5	0.6 × 0.6
Nott_103c	Siemens	Aera	1.5	00006-MR-resolve_4scan_trace_tra_160_p2_DWI_TRACEW	60	6780	4	4.96	1.6 × 1.6
Nott_109a	GE Medical Systems	Discovery MR750w	3	00003-MR-DWI_TRA_b1250	75	12025	3.6	3.9	0.9 × 0.9
Nott_109b	Siemens	Aera	1.5	00005-MR-ep2d_diff_3scan_trace_p2_TRACEW	89	7800	4	5	0.6 × 0.6
Nott_111a	Philips	Achieva	3	00601-MR-DWI	95	4442	4	4.4	0.9 × 0.9
Nott_111b	Siemens	Aera	1.5	00005-MR-ep2d_diff_3scan_trace_p2_TRACEW	89	7800	4	5	0.6 × 0.6
Nott_111c	Siemens	Aera	1.5	00005-MR-ep2d_diff_3scan_trace_p2_TRACEW	89	8100	4	5	0.6 × 0.6
Nott_116a	Philips	Achieva	3	00601-MR-DWI	95	4032	4	4.4	0.9 × 0.9
Nott_116b	Siemens	Aera	1.5	00005-MR-ep2d_diff_3scan_trace_p2_TRACEW	89	8600	4	5	0.6 × 0.6
Nott_120a	Siemens	Aera	1.5	00005-MR-ep2d_diff_3scan_trace_p2_TRACEW	115	6700	4	4.4	1.3 × 1.3
Nott_120b	Siemens	Aera	1.5	00005-MR-ep2d_diff_3scan_trace_p2_TRACEW	89	7800	4	5	0.6 × 0.6
Nott_120c	Philips	Ingenia	1.5	00401-MR-DWI	90	3627	4	5	1.3 × 1.3

## Data Availability

The data presented in this study are available on request from the corresponding author. The data are not publicly available due to ongoing research studies.
